# Translating bed total body irradiation lung shielding and dose optimization using asymmetric MLC apertures

**DOI:** 10.1120/jacmp.v17i2.5951

**Published:** 2016-03-08

**Authors:** Shahbaz Ahmed, Derek Brown, Saad B. S. Ahmed, Muhammad B. Kakakhel, Wazir Muhammad, Amjad Hussain

**Affiliations:** ^1^ Department of Physics and Applied Mathematics (DPAM) Pakistan Institute of Engineering and Applied Sciences (PIEAS) Islamabad Pakistan; ^2^ Department of Radiation Medicine and Applied Sciences University of California San Diego La Jolla CA; ^3^ Department of Oncology Aga Khan University Hospital Karachi Pakistan; ^4^ Health Physics Division (HPD) Pakistan Institute of Nuclear Sciences and Technology (PINSTECH) Islamabad Pakistan

**Keywords:** total body irradiation, asymmetric MLC apertures, dynamic MLC, lung shielding, dose optimization

## Abstract

A revised translating bed total body irradiation (TBI) technique is developed for shielding organs at risk (lungs) to tolerance dose limits, and optimizing dose distribution in three dimensions (3D) using an asymmetrically‐adjusted, dynamic multileaf collimator. We present a dosimetric comparison of this technique with a previously developed symmetric MLC‐based TBI technique. An anthropomorphic RANDO phantom is CT scanned with 3 mm slice thickness. Radiological depths (RD) are calculated on individual CT slices along the divergent ray lines. Asymmetric MLC apertures are defined every 9 mm over the phantom length in the craniocaudal direction. Individual asymmetric MLC leaf positions are optimized based on RD values of all slices for uniform dose distributions. Dose calculations are performed in the Eclipse treatment planning system over these optimized MLC apertures. Dose uniformity along midline of the RANDO phantom is within the confidence limit (CL) of 2.1% (with a confidence probability p=0.065). The issue of over‐ and underdose at the interfaces that is observed when symmetric MLC apertures are used is reduced from more than ±4% to less than ±1.5% with asymmetric MLC apertures. Lungs are shielded by 20%, 30%, and 40% of the prescribed dose by adjusting the MLC apertures. Dose‐volume histogram analysis confirms that the revised technique provides effective lung shielding, as well as a homogeneous dose coverage to the whole body. The asymmetric technique also reduces hot and cold spots at lung‐tissue interfaces compared to previous symmetric MLC‐based TBI technique. MLC‐based shielding of OARs eliminates the need to fabricate and setup cumbersome patient‐specific physical blocks.

PACS number(s): 87.55.‐x, 87.55.de, 87.55.D‐

## I. INTRODUCTION

Unlike conventional radiotherapy where part of the body is irradiated, total body irradiation (TBI) is a specialized radiotherapy technique where the patient is exposed to high whole‐body doses of radiation. TBI is usually prescribed for: a) suppressing the immune system of the patient prior to stem cell transplant, or b) treating various hematological diseases (i.e., leukemia, lymphoma, anemia).^(1)^ Large radiation fields are required to cover the entire patient during TBI dose delivery. In some cases, dedicated and specialized TBI facilities are used to cover the entire patient body.^2^, ^3^, ^4^, ^5^, ^6^ To limit doses to OARs, labor‐intensive physical shielding blocks are usually used.^(7)^

Several groups have developed dynamic TBI techniques for irradiating the entire patient by moving either the patient or the radiation source. Constant and variable bed velocity TBI, aperture modulated TBI, and radiological depth‐based TBI are a few examples of dynamic TBI techniques.^1^, ^4^, ^5^, ^8^, ^9^ Some other techniques suggest the use of sweeping arc fields similar to volumetric‐modulated arc therapy (VMAT) techniques.^10^, ^11^

One of the complications in the implementation of dynamic TBI techniques is that commercially available treatment planning systems (TPS) are designed to perform dose calculations for relatively small volumes, at nominal source‐to‐axis distance (SAD). In TBI however, the beam arrangements and patient positioning are significantly different from this standard. Recently a novel radiological depth (RD)‐based TBI technique (RD‐TBI) has been designed for improving dose distribution uniformity by translating the patient under a radiation beam while dynamically optimizing MLC apertures.^(9)^ We refer to this method as Symmetric MLC‐based Technique for TBI (SMT‐TBI). The SMT‐TBI technique uses symmetric MLC apertures for dose optimization that can deliver a uniform radiation dose to within ∼±4% of the prescribed dose. This method results in improved overall dose uniformity and accuracy compared to static TBI techniques, but nevertheless produces regions of over‐ and underdose in parts of the body with sharp changes in RD (e.g., lung‐tissue interfaces).^(9)^

In SMT‐TBI, the MLC leaf positions are defined using radiological depth (RD) values from the CT slice at the level of the beam central axis.^(9)^ Both leading and trailing MLC leaves are symmetrically positioned about the central axis. The average planning slice thickness (distance between two adjacent MLC apertures [e.g., 0.9 cm]) is much less than the beam aperture length (e.g., 8‐12 cm at source‐to‐table distance STD of 200 cm). Therefore, an individual MLC aperture nearly irradiates 9 to 15 planning CT slices. This implies that a single slice receives an integrated dose according to the RD values of multiple neighboring slices which might have different RD values and, therefore, a different aperture value. This results in dose deviation in other slices with RD values different than that of the slice at central axis. This effect is more pronounced at tissue interfaces where sharp RD changes occur, such as head and neck and thoracic region. Furthermore, with the SMT‐TBI technique no mechanism for lung shielding to a specified dose limit is provided and shielding can only be achieved at the expense of large underdose of normal tissue at the lung tissue interface.

The current study presents a modified SMT‐TBI technique that uses asymmetrically defined MLC apertures to account for the sharp RD changes and is referred to as Asymmetric MLC‐based Technique (AMT‐TBI). We investigate the feasibility and accuracy of lung shielding with this revised technique, aiming to avoid the use of heavy physical shielding blocks, which are time‐consuming and labor‐intensive to design and fabricate.^6^, ^12^, ^13^

## II. MATERIALS AND METHODS

### A. CT simulation and RD calculation

A whole body CT scan of a 99 cm long anthropomorphic RANDO phantom (The Phantom Laboratory, Salem, NY) is performed with a 3 mm slice thickness. CT images are imported into MATLAB version R2013a (Math Works, Inc., Natick, MA), for radiological depth (RD) calculation (discussed in Hussain et al.^(9)^) using the following relation:

(1) $$RD=\frac{\sum_{i=1}^{n}d_{i}\rho_{i}}{\rho_{water}}cm$$

where $d_{i}$ denotes the physical thickness of the tissue segment with a uniform electron density $\left(\rho_{i}\right)$, and *n* is the total number of such tissue segments along the ray line, incorporating the beam divergence.

### B. MLC aperture size calibration

**Figure 1:**
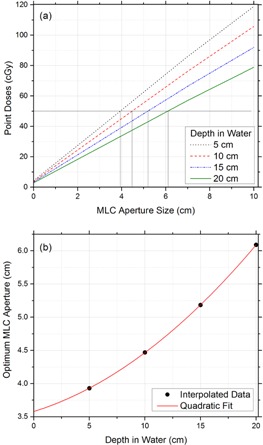
Calculated point doses (a) versus MLC aperture size for different RD values; optimum MLC aperture (b) versus RD.

To deliver a uniform radiation dose to the entire patient body in a translating bed technique, optimum aperture shapes need to be designed along different body sections in conjunction with the translating bed. (The reader is referred to Hussain et al.^(9)^ for further technical details.) The beam apertures need to be wider for radiologically thicker sections of the phantom/patient, and vice versa. The calibration is performed with the same method as RD‐TBI, but at a source‐to‐table distance (STD) of 200 cm, instead of 204.5 cm as in RD‐TBI. Rectangular water phantoms (30 cm wide, 100 cm long, 15‐30 cm deep) were created in Eclipse (Varian Medical Systems, Palo Alto, CA) and were virtually translated under the rectangular MLC fields with preset aperture value ranging from 0 to 10 cm (with X jaw fixed at 10 cm and Y jaw at 30 cm). The virtual translation is achieved by defining multiple fields along the length of the phantom. Each field is separated by 1 cm from adjacent fields. Point doses were calculated at different depths (5, 10, 15, and 20 cm) in water phantom. Each field was delivered 20 MUs, which corresponds to 15 cm/min couch speed and 300 MU/min dose rate. These calculated point doses are plotted against the aperture values for different depths in Fig. 1(a). A quadratic relation between RD and beam aperture length is derived using Eq. (2) and is shown in Fig. 1(b). Treating with parallel opposed beams, the apertures are optimized to deliver a 100 cGy prescribed dose per fraction.

(2) $$Optimum\;Aperture\;Size=0.0037\;RD^{2}+0.05\;RD+3.6$$

### C. Dynamic MLC‐based lung shielding

The aperture optimization is carried out using the same method as described above; however, the beam apertures over the lungs are further adjusted per shielding requirements. Desired percentage shielding, or in other words, the reduction in lung dose as compared to the prescription is termed as shielding fraction S. The apertures are modified by a shielding scale factor F. This (F) is defined as the ratio of MLC aperture values for shielding fraction S and the MLC aperture values for zero shielding. The MLC aperture values for shielding fraction S are derived from Fig. 1(a) (e.g., 20%, 25% and so on up to 50% [this corresponds to 80%, 75%, and 50% transmission, respectively]). A relation between shielding scale factor F, RD, and S is derived by surface fitting using least squares optimization with the help of ThreeDify Excel Grapher Add‐In in Microsoft Excel (Microsoft Inc., Redmond, WA):

(3) $$F=3\times 10^{-5}\times RD^{2}-1.085S+1$$

**Figure 2:**
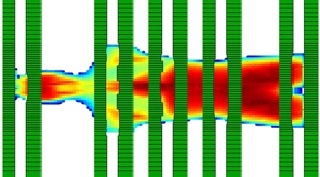
Beam's eye view of some of the selected asymmetric MLC apertures over the RANDO.

A schematic representation is given in Fig. 2 for some selected MLC apertures over the patient at different time intervals, with 20% (i.e., S=0.20) lung shielding. The RD values for the lung region, as expected, are lower than the surrounding values. This region is contoured in MATLAB and the RD values are scaled by the scaling factor F calculated for the desired lung shielding. The study is performed for 20%, 30%, and 40% lung shielding with the corresponding ‘F’ scaling factors of 0.784, 0.676, and 0.568, respectively.

### D. Aperture modulation

In the AMT‐TBI technique, the leading and trailing MLC leaf positions are optimized such that each point along the craniocaudal axis remains in the radiation field for a time period that results in a desired uniform dose to all these points. This means that the previous and subsequent beam apertures around the central axis are adjusted such that the CT slice at the central axis is exposed to the beam for a time period that is optimized for this slice. The entire aperture optimization process is performed using an in‐house MATLAB code. (The authors can be contacted by email for obtaining code files.) The optimization algorithm looks to the nearest neighbor planning CT slices relative to the slice of interest and asymmetrically optimizes the MLC positions of the neighboring apertures. In this way, the MLC aperture value is optimized for all the planning CT slices being irradiated. In the symmetric MLC technique, the MLC aperture values are symmetrically shaped according to the RD value of the slice at the central axis only.

A total of 119 (10+99+10) fields are defined to cover the entire RANDO phantom. In order for the leading and trailing MLCs to reach the intended positions and cover the entire phantom, treatment fields are extended 10 cm beyond RANDO on either side. Individual static MLC files, with a craniocaudal step size of 9 mm are created for dose calculation purposes. The complete procedure is automated in MATLAB. For treatment delivery, a single dynamic MLC file is created, which is to be delivered with the translating bed under the radiation field.

### E. Dose calculations and treatment strategy

The optimized individual static MLC files are imported into the Eclipse treatment planning system for dose calculation with a 9 mm interval in between two consecutive apertures along the craniocaudal direction. Dose calculations are performed by the analytical anisotropic algorithm (AAA) version 10.1 in Eclipse, using photon energy of 6 MV at extended source‐to‐table distance (STD) of 200 cm and machine dose rate of 300 MU/min (effective dose rate at the patient body ∼4 cGy/min) with heterogeneity corrections turned on. This along with the incorporation of scatter corrections within the RD calculations inherently takes into account the lung dose correction factors, as reported earlier.^14^, ^15^ Supine delivery is modeled using beams at a gantry angle of 0° on the supine CT scan. Prone delivery is modeled using the same CT scan, but with beams at a gantry angle of 180°. A collimator rotation of 270° orients the MLCs correctly to model the cross‐plane movement of the translating bed.^(9)^ X jaw positions along MLC were adjusted to 10 cm, whereas Y jaws were 30 cm wide along lateral dimensions of the RANDO. Dose calculations in Eclipse using AAA algorithm has already been validated in a previous study.^(16)^ Moreover, in a similar study published earlier, dose delivery accuracy has been verified using TLDs and Gafchromic EBT films (International Specialty Products, Wayne, NJ) in a RANDO phantom.^(8)^ Both point doses in a number of positions and dose profiles were compared and a deviation of ±3% was observed.^(8)^ Since there is no major change in the dose delivery mechanism, the previous treatment procedure and dose verification methodology is assumed valid for the current scenario, as well.^8^, ^9^ A quality assurance procedure to ensure proper correlation between MLC leaf positions and bed position has been suggested by Granville and Brown in an abstract presented at the AAPM annual meeting 2011.^(17)^

## III. RESULTS

The 3D dose distribution is analyzed by drawing line dose profiles through various body sections, as well as using color wash to visualize dose discrepancies. Dose‐volume histograms are also presented. The concept of confidence limit (CL) is employed to represent a true picture of midline dose uniformity, by incorporating the mean and standard deviation (SD) of dose distribution. The confidence limit, as defined by Venselaar et al.,^(18)^ is calculated as:

(4) CL=|Mean Deviation|+1.5×Standard Deviation

**Figure 3:**
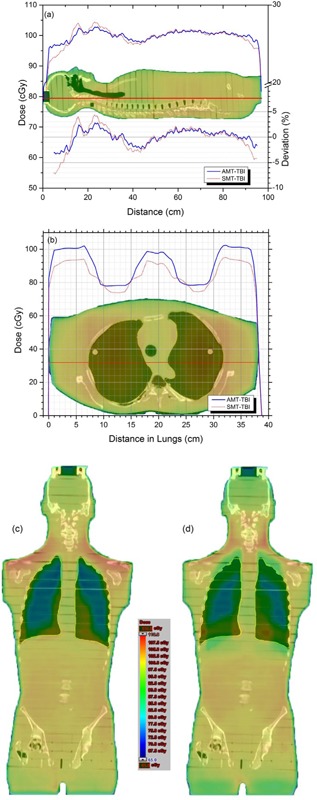
Midline dose profiles (a), transverse lung dose profile (b), and frontal dose distributions with (c) AMT‐TBI and (d) SMT‐TBI (each with shielding scale factor 0.676).

A midline dose profile in the superior‐inferior direction is shown in Fig. 3(a) for AMT‐TBI versus SMT‐TBI. For AMT‐TBI, dose deviation from prescription is determined to be well within a confidence limit of 2.1% (confidence probability p=0.065). The results are significantly better as compared to SMT‐TBI, where a 3.0% confidence limit is observed. Dose distributions in color shades are presented in Figs. 3(c) and (d). Overall, dose distribution with AMT‐TBI is more uniform compared to the SMT‐TBI; however, shoulders are a bit hotter in the AMT‐TBI technique.

**Figure 4:**
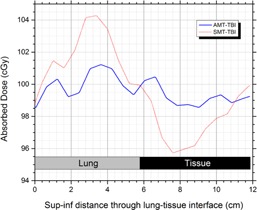
Vertical dose profile through the lung with no lung shielding.

**Figure 5:**
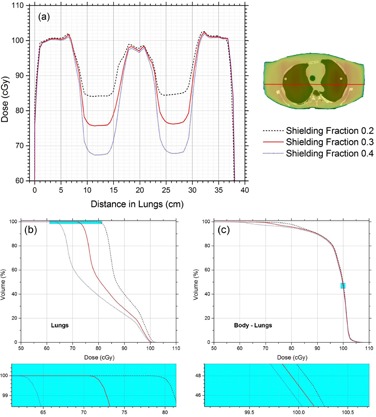
Line dose profiles (a) through lungs, (b) DVH for lungs, and (c) DVH for body volume.

The over‐ and underdose at the lung‐tissue interface is graphically shown in Fig. 4 by drawing a vertical dose profile through the interface. It is clear that at this position, dose inhomogeneity is of the order of ±4% with SMT‐TBI, whereas the deviation is significantly reduced to ±1.5% with the AMT‐TBI methodology. Dose profiles in the transverse dimension through the lungs are presented in Fig. 5(a) for AMT‐TBI with three shielding fractions (0.2, 0.3, and 0.4) excluding lungs, all with AMT‐TBI with lung shielding. The profiles indicate that lungs are effectively shielded to different dose levels, without compromising dose in the adjacent soft tissue in craniocaudal and lateral dimensions, as shown in Figs. 3(b), (c), and (d). Per the dose‐volume histogram (Fig. 5(b)), 100% of the lungs volume receives 62%, 71%, and 79% of prescribed dose when shielded to 40%, 30%, and 20%, respectively, with AMT‐TBI. It is clear from the DVH in Fig. 5(c) that the effect of the lungs shielding on the dose to the rest of the body volume is insignificant. The body volume covered by 90% of the prescription dose is approximately equal to or more than 90% in all cases. Various dosimetric parameters ($V_{D}$: percent volume receiving dose D, and $D_{V}$: dose received by percent volume) are presented in Table 1 for lungs and whole‐body volume excluding lungs. The data are presented for AMT‐TBI only, with different shielding fractions.

**Table 1:** Different dosimetric parameters ($V_{D}(\%)$ and $D_{V\%}$) for lungs and whole body volume excluding lungs, treated with AMT‐TBI using different shielding fractions

	$V_{D}(\%)$ *for Lungs*		
*SF*	$V_{60\text{cGy}}$	$V_{70\text{cGy}}$	$V_{80\text{cGy}}$	$V_{90\text{cGy}}$
0.2	100	100	100	40.0
0.3	100	100	52.5	28.0
0.4	100	60.0	38.0	23.4
	$D_{V\%}$ *(cGy) for Body Excluding Lungs*		
*SF*	$D_{80\%}$	$D_{90\%}$	$D_{95\%}$	$D_{100\%}$
0.2	95.9	90.1	84.0	69.0
0.3	95.5	90.0	81.8	65.0
0.4	95.6	89.2	80.0	60.0

SF=shielding fraction.

## IV. DISCUSSION

The results (Figs. 3, 4, 5) demonstrate that the AMT‐TBI technique is almost equivalent to SMT‐TBI technique in terms of dose homogeneity and superior regarding lung shielding. This is supported by the improved dose uniformity at the tissue‐lung interface where there are abrupt changes in RD values and confirms the importance and uniqueness of AMT‐TBI technique for lungs shielding.

It has also been established in the literature that in order to achieve improved dose sparing for lungs, it is more important to reduce the lower doses to larger lung volume than to reduce the higher doses to lesser volume.^11^, ^19^ This has been achieved using AMT‐TBI which successfully manipulates lung doses to recommended tolerance levels. Not only this, but the absorbed dose to the remaining body is not compromised with lung shielding. Some of the body volume in the thoracic region (above and below lung region along beam direction) receives lower doses due to lung shielding. Dose to this volume can be supplemented by applying electron boost.^20^, ^21^, ^22^

For the first few centimeters towards the head of the phantom, midline dose is considerably lower due to the presence of an air cavity inherent to the construction of the RANDO phantom. On logical grounds, the remaining dose perturbation observed with AMT‐TBI may be attributed to three factors, namely: internal inhomogeneities, contour variations, and difference in scatter distributions. Sharp contour variations in the head and neck region lead to sharp RD changes in those regions. However, AMT‐TBI has shown better dose homogeneity in these regions than that achievable with SMT‐TBI (Fig. 3(a)). A decreased absorbed dose in the head region may be attributed to the lack of back scatter. Further, a higher absorbed dose in the neck region may be attributed to the presence of oral cavity and respiratory air pathways, causing scatter disruptions. However, with AMT‐TBI the overall dose homogeneity is better in these regions as compared to that with SMT‐TBI, as shown in Figs. 3(c) and (d). Absorbed dose in the shoulders is relatively higher (108% of the prescribed dose) with the AMT‐TBI. A possible reason is the abrupt change in RD value at air‐tissue interface. Aperture optimization is somehow compromised at this point with the AMT‐TBI as opposed to SMT‐TBI where this higher dose is compensated by the following MLC apertures designed for lung region only.

In the thoracic region, the midline dose is lowered due to the presence of lungs.^(14)^ The tissues adjacent to the lungs receive less scatter dose because of the lower density of lungs. A significant under‐ and overdose is observed around the lung‐tissue interfaces with the SMT‐TBI. However, AMT‐TBI ensures better dose homogeneity at the interfaces, confirming the hypothesis presented in the Introduction section, and is validated by the dose profiles presented in Fig. 4. The dose coverage for the whole body excluding lungs is better with AMT‐TBI even with lung shielding incorporated (Figs. 5(b) and (c), and Table 1).

The results obtained with this revised technique are promising and superior to the SMT‐TBI translating bed technique in terms of lung shielding and dose homogeneity.^2^, ^3^, ^4^, ^5^, ^8^, ^9^ Respiratory motion management is an important consideration in radiotherapy treatments. In the case of translating bed techniques, the dose distribution will blur at the lung‐tissue interfaces as a result of the patient breathing or coughing.^(23)^ A possible solution to this limitation is the use of breath‐hold procedures. In addition, other unintended patient motions are difficult, if not impossible, to account for with translating bed techniques. The implementation of real‐time positional monitoring, such as optical surface monitoring, may be a solution to ensuring *in vivo* positional accuracy — a possibility worthy of future study. Finally, it is worth noting that overall treatment planning and delivery time is a bit longer for this technique.

## V. CONCLUSIONS

With the revised AMT‐TBI technique, effective lung shielding to a specified dose limit can be achieved without compromising the 3D dose homogeneity (midline dose within confidence limit of 2.1%) in a RANDO phantom. Furthermore, the time‐consuming and laborious procedure of designing and fabrication of heavy, patient‐specific shielding blocks for translational TBI may be avoided.

## ACKNOWLEDGMENTS

The authors acknowledge the financial and infrastructural support provided by Pakistan Institute of Engineering & Applied Sciences (PIEAS), Islamabad and Aga Khan University Hospital (AKUH), Karachi during the course of this research project.

## COPYRIGHT

This work is licensed under a Creative Commons Attribution 4.0 International License.

## Supporting information

Supplementary Material Files

Supplementary Material Files

Supplementary Material Files

Supplementary Material Files
