# Supplementary material for: Translating bed total body irradiation lung shielding and dose optimization using asymmetric MLC apertures
Source: J Appl Clin Med Phys. 2016 Mar 8;17(2):112–22. doi: 10.1120/jacmp.v17i2.5951 (PMC5875554; doi:10.1120/jacmp.v17i2.5951)
Supplement: Supplementary file 4 — Supplementary Material Files [file ACM2-17-112-s004.pdf]

Medical Physics
The International Journal of Medical Physics Research and Practice

SU-C-BRB-02: Symmetric and Asymmetric MLC Based Lung Shielding and Dose Optimization During Translating Bed TBI

S Ahmed[1], MB Kakakhel[1], SBS Ahmed[2] and A Hussain[2]
Med. Phys. 42, 3193 (2015); http://dx.doi.org/10.1118/1.4923805

Abstract

Purpose:
The primary aim was to introduce a dose optimization method for translating bed total body irradiation technique that ensures lung shielding dynamically. Symmetric and asymmetric dynamic MLC apertures were employed for this purpose.

Methods:
The MLC aperture sizes were defined based on the radiological depth values along the divergent ray lines passing through the individual CT slices. Based on these RD values, asymmetrically shaped MLC apertures were defined every 9 mm of the phantom in superior-inferior direction. Individual MLC files were created with MATLAB™ and were imported into Eclipse™ treatment planning system for dose calculations. Lungs can be shielded to an optimum level by reducing the MLC aperture width over the lungs. The process was repeated with symmetrically shaped apertures.

Results:
Dose-volume histogram (DVH) analysis shows that the asymmetric MLC based technique provides better dose coverage to the body and optimum shielding of the lungs compared to symmetrically shaped beam apertures. Midline dose homogeneity is within ±3% with asymmetric MLC apertures whereas it remains within ±4.5% with symmetric ones (except head region where it drops down to −7%). The substantial over and under dosage of ±5% at tissue interfaces has been reduced to ±2% with asymmetric MLC technique. Lungs dose can be reduced to any desired limit. In this experiment lungs dose was reduced to 80% of the prescribed dose, as was desired.

Conclusion:
The novel asymmetric MLC based technique assures optimum shielding of OARs (e.g. lungs) and better 3-D dose homogeneity and body-dose coverage in comparison with the symmetric MLC aperture optimization.

The authors acknowledge the financial and infrastructural support provided by Pakistan Institute of Engineering & Applied Sciences (PIEAS), Islamabad and Aga Khan University Hospital (AKUH), Karachi during the course of this research project. Authors have no conflict of interest with any national / international body for the presented work.

© 2015 American Association of Physicists in Medicine
DOI: http://dx.doi.org/10.1118/1.4923805
